# On the Utility of the Oxygenated Muriatic Acid in the Cure of Scarlet Fever, *with an Easy Mode of Preparing It for Medical Purposes*

**Published:** 1804-04-01

**Authors:** John Ayrey Brathwaite

**Affiliations:** Member of the Royal College of Surgeons, in London, and Surgeon to the Lancaster Dispensary


Medical and Phyſical Journal.

VOL. XI.] APRIL 1, 1804. [NO. LXII.

*Printed for R. PHILLIPS, by W. Thorne, Red Lion Court, Fleet Street, London.*

### ON THE UTILITY OF THE OXYGENATED MURIATIC ACID IN THE CURE OF SCARLET FEVER, *with an easy Mode of preparing it for Medical Purposes*; *by* JOHN AYREY BRATHWAITE, *Member of the Royal College of Surgeons, in London, and Surgeon to the Lancaster Dispensary.*

HAVING frequently experienced the inefficacy of the common mode of medical practice in the scarlatina anginosa, I have been induced to make some inquiries into the nature, cause, and treatment of that disease, which has been prevalent in this town and neighbourhood for three years last past; the result of my observations have been the discovery of a remedy in this disease, which is as much entitled to infallibility as mercury in the lues, or bark in the ague; it is easily prepared by any apothecary, of materials with which his shop is or ought to be always supplied, and requires no complex pharmaceutical apparatus, by which often those unaccustomed to practical chemistry are liable, even from proper materials, to prepare chemical preparations totally different in their properties from those intended.

As I have no doubt but the contagion of the scarlet fever produces an extraordinary degree of dis-oxygenation of the system, with great debility, and exhaustion of the sensorial power, I was led to suppose that oxygen, exhibited in some easy and pleasant manner, might not only destroy the contagious matter adhering to the tonsils, uvula, &c. but by penetrating the fine moist membrane of the lungs, and by chemical attraction uniting with the blood, excite the action of the arterial system, warming the extremities, increasing insensible perspiration, exhilirating the spirits, and invigorating the vital principle without exhausting it, would prove an efficacious remedy in this but too fatal disease. This I have experienced in the oxygenated muriatic acid, whose known property of destroying putrid miasms, and preventing infection, in a gaseous state, has totally abolished the absurd farragos of antient practice.

Variolous and vaccine virus exposed but for a moment to the vapour of oxygenated muriatic acid, lose their contagious properties, and the latter rubbed with one eighth of a grain of oxyde of iron *(rubigo ferri)* will rarely communicate the disease; what then may we not expect from this active and elegant preparation? Elegant I may justly intitle it, as, when properly prepared and sufficiently diluted, it may be administered to patients of all ages, being a safe and efficacious remedy, possessed of a slight degree of grateful acidity.

When called to a patient in whatever stage of the scarlet fever, my practice for two years last past has uniformly been as follows: One drachm of oxygenated muriatic acid is mixed with eight ounces of distilled water in a phial and shaken together; this quantity should be taken every twelve hours, by a patient from fourteen to twenty years of age, but it is preferable to administer it in draughts divided from the quantity above mentioned, into ℥ij. ℥jß. ℥j. and ℥ß. bottles, as the patient's age and situation require, ordering them to be taken at such periods as for an adult to consume the quantity in the time mentioned, and to younger patients smaller doses, as half a drachm or two scruples of the acid to eight ounces of water. By this method, the oxygen gas is not separated and lost, each time the phial is opened, as may easily be perceived by its smell in the apartment; it is also absolutely necessary the medicine be placed in a dark situation, wrapped in paper, to prevent the dis-oxygenating influence of light.

Since the use of this medicine, I have never had recourse to emetics, purgatives, blisters or diaphoretics, a regular perseverance in the oxygenant remedy has universally succeeded, my patients rapidly recovering, and being seldom afflicted with those complaints succeeding the scarlet fever, such as pain of the joints, paucity of urine, and universal anasarcous swellings; even should these follow, I recommend a continuance of the medicine, until these symptoms entirely disappear, which will be found much earlier than by the usual mode of treatment; indeed, if the oxygenated preparation is duly persevered in, I am of opinion those painful and distressing affections will rarely occur. It is also possessed of this desirable property, that it may be easily taken by children, who generally are the most numerous patients in this disease, and to whom all medicines medicines are administered with difficulty. I have heard them frequently cry for *that stuff which mended their throat*, as they expressed it; indeed, in that respect, its effects are truly admirable, far surpassing the disagreeable practice of gargling and syringing, which in numerous instances, even if possible to do it, is productive of mischief; how far superior then must be a remedy which by passing over the infected and frequently ulcerated part immediately, not only gives instantaneous relief, but entirely removes that fœtid smell originating in several cases from these parts? Patients often wish to be frequently sipping a little of the oxygenant liquid, which is not improper, but it must be always done out of a wine glass, as admeasurement with a spoon is dangerous, the oxygen rapidly oxydating the metal of which it is composed, and by that means conveying into the stomach a poisonous fluid, from which death might ensue.

The muriatic acid has long been used as a medicine, and Sir William Fordyce strongly recommended it in the ulcerated sore throat and putrid fever, but the oxygenated muriatic acid has, I believe, been rarely employed. Dr. Crawford* once took twenty drops of it diluted with water, but soon afterwards found an obtuse pain with a sensation of constriction in the stomach and bowels; this uneasiness, notwithstanding the use of emetics and purgatives, lasted for several days, and was at last removed by drinking water impregnated with sulphureous hepatic air; this effect he attributes to the manganese which had been used in the distillation of the acid, containing a portion of lead; I should rather suppose it proceeded from the dose of twenty drops being taken; oxygenated muriatic acid readily gives to living animal bodies its super-oxygen, and the remains are common muriatic acid, a dose of which, similar to the above, would undoubtedly in delicate constitutions produce similar effects. In no case whatsoever have I found it necessary to exceed the quantity before mentioned, but it has sometimes been done by my patients, through an anxious desire to get well; the same uneasiness has however been produced as mentioned by Dr. Crawford, though the preparation was made so as not possibly to contain either lead or any other metallic substance.

To prepare the oxygenated muriatic acid in a perfect state of purity; put two ounces by measure of distilled

* Philosoph. Trans. vol. 80.

water into a narrow tubulated bottle, with a ground glass stopple; into this gradually pour, by measure also, as much muriatic acid, the specific gravity of which is as 1170 to 1000 of distilled water, frequently shaking the phial; add then to it two drachms of oxy-muriate of pot-ash,* which in a little time will fall to the bottom, the acid seizing the small portion of alkali, and leaving beautiful globules of vital air, which rise slowly towards the surface, diminishing as they ascend, super-oxygenating the acid; a little agitation now and then facilitates the process, but it will be three or four days before the acid becomes extra-oxygenated; the stopple should be put loosely into the phial, and tied over with a piece of bladder, but not too tight, allowing it to move when the gas is rapidly extricated; this process should be performed in a dark situation, and the oxygenant medicine after preserved by putting over the bottle a circular piece of pasteboard, to prevent it from being injured by the dis-oxygenating power of light.

It is not in scarlet fever only that this preparation promises to be of advantage, I have found it useful in angina maligna, and other diseases proceeding from or producing a dis-oxygenation of the blood; in many lingering cases of the late influenza, it was exhibited with evident advantage in the doses above mentioned.

From the trials made by Guyton Morveau and others, it appears that the oxygenated muriatic acid, in a gaseous form, possesses the power of neutralizing and destroying contagious miasmata, even in rooms where the sick are present, without the slightest inconvenience; possessed of amazing expansibility, this gaseous oxygenant diffuses itself over the most extensive apartments, leaving nothing untouched, and touching nothing it does not appropriate, rapidly oxydating metallic bodies, particularly iron and steel, which should be removed, and radically destroying the most offensive odours, thereby rendering innocuous perhaps deadly contagious poisons.

To completely purify any apartment when a patient suffers in the scarlet fever, or any other contagious disease, so as to render it perfectly safe, not only to the attendants but to the rest of the family, take a china tea cup and saucer; put in the cup two ounces of common salt, and half an

---

* The best oxy-muriate of pot-ash I ever had, was made by Mr. Hoyle, an ingenious chemist in Manchester, 100 grains yielding nearly 74 cubic inches of oxygen gas.

an ounce of the black oxyd of manganese, previously powdered with one ounce of water; then take an ounce and a half of sulphuric acid, and pour a little of it now and then into the tea cup, among the other ingredients; immediately an amazing quantity of oxygenated muriatic acid gas will be disengaged and fill the apartment; this should be suffered to remain only a few minutes, removing it out of the room into the stair-case, by which means the whole house will be become impregnated with this gaseous oxygenant; it will be proper to take it into the room frequently during the day, adding to it a little fresh sulphuric acid, and then replacing it in its former situation.

It was my intention to have transmitted a more minute account of the scarlet fever, and its mode of treatment by this oxygenant remedy, illustrated with cases; but suffering at present under an arthritic complaint, I found myself inadequate to the undertaking; perhaps, at some future period, I may again take up the pen to corroborate what I have asserted; should this, however, be the means of rescuing one individual from a premature grave, the intention of the writer will, in some degree, be accomplished.

*Lancaster, March* 1, 1804.

